# Factors influencing sustainability and scale-up of rural primary healthcare memory clinics: perspectives of clinic team members

**DOI:** 10.1186/s12913-022-07550-0

**Published:** 2022-02-04

**Authors:** Debra Morgan, Julie Kosteniuk, Megan E. O’Connell, Dallas Seitz, Valerie Elliot, Melanie Bayly, Amanda Froehlich Chow, Chelsie Cameron

**Affiliations:** 1 Canadian Centre for Health & Safety in Agriculture, University of Saskatchewan, 104 Clinic Place, Saskatoon, SK S7N 2Z4 Canada; 2 Department of Psychology, University of Saskatchewan, Arts 182, 9 Campus Drive, Saskatoon, SK S7N 5A5 Canada; 3 Department of Psychiatry, Hotchkiss Brain Institute, and O’Brien Institute for Public Health, Cumming School of Medicine, University of Calgary, Room 2919 Health Sciences Centre, 3330 Hospital Drive NW, Calgary, AB T2N 4N1 Canada; 4 School of Public Health, University of Saskatchewan, 104 Clinic Place, Saskatoon, SK S7N 2Z4 Canada

**Keywords:** Sustainability, Spread, Scaling up, Primary healthcare, Memory clinic, Rural, Dementia

## Abstract

**Background:**

The aging of rural populations contributes to growing numbers of people with dementia in rural areas. Despite the key role of primary healthcare in rural settings there is limited research on effective models for dementia care, or evidence on sustaining and scaling them. The purpose of this study was to identify factors influencing sustainability and scale-up of rural primary care based memory clinics from the perspective of healthcare providers involved in their design and delivery.

**Methods:**

Participants were members of four interdisciplinary rural memory clinic teams in the Canadian province of Saskatchewan. A qualitative cross-sectional and retrospective study design was conducted. Data were collected via 6 focus groups (*n* = 40) and 16 workgroup meetings held with teams over 1 year post-implementation (*n* = 100). An inductive thematic analysis was used to identify themes.

**Results:**

Eleven themes were identified (five that influenced both sustainability and scale-up, three related to sustainability, and three related to scale-up), encompassing team, organizational, and intervention-based factors. Factors that influenced both sustainability and scale-up were positive outcomes for patients and families, access to well-developed clinic processes and tools, a confident clinic leader-champion, facilitation by local facilitators and the researchers, and organizational and leadership support. Study findings revealed the importance of particular factors in the rural context, including facilitation to support team activities, a proven ready-to-use model, continuity of team members, and mentoring.

**Conclusions:**

Interdisciplinary models of dementia care are feasible in rural settings if the right conditions and supports are maintained. Team-based factors were key to sustaining and scaling the innovation.

**Supplementary Information:**

The online version contains supplementary material available at 10.1186/s12913-022-07550-0.

## Introduction

The expanding field of implementation science reflects increasing awareness that many innovative pilot programs do not achieve their full policy and program impact because of challenges in sustainability and scaling up [1]. Early efforts to address this research-to-practice gap focused on understanding factors influencing successful initial implementation of evidence-based innovations [2]. Recognition that not all interventions that have been successfully implemented are continued has highlighted the need to examine sustainability as a separate phenomenon. The gap between evidence and practice is particularly acute with respect to dementia care [3, 4], exacerbated by the challenges of delivering dementia services in rural settings [5–7]. This paper begins to address these identified gaps by focusing on factors influencing the sustainability and scale-up of a primary healthcare innovation for dementia care in a rural setting.

Studying sustainability has been hampered by lack of conceptual clarity and variation in operational definitions [8, 9]. Early views of sustainability as an end stage of the implementation process have moved toward the idea of a change process of continuous intervention improvement [10, 11]. For example, the Dynamic Sustainability Framework [12] links sustainability to ongoing adaptation aimed at responding to system needs and improving intervention-context fit. The concept analysis by Fleiszer et al. [13] identified three main elements: continued benefits, persistence of the innovation, and ongoing innovation development. Reported influences on sustainability include characteristics of the innovation and the individuals involved, leadership, capacity, processes, and context [2, 9, 13, 14].

The language used to refer to expanding the reach of innovations is also used inconsistently. While the terms spread and scale-up are often used interchangeably, others have noted that “spread” typically refers to passive diffusion of local innovations within a setting while “scale-up” suggests a systematic roll-out on a larger scale [8, 15]. The term “horizontal scale-up” has been used to describe a phased approach and “vertical scale-up” to simultaneous implementation in a whole system [8]. In a similar vein, the World Health Organization/ExpandNet [16] differentiates “horizontal scale-up” (replication in different geographic sites or populations) from “vertical scale-up” (formal government adoption of an innovation). The evidence regarding strategies for scaling up and spreading is even more limited than for sustainability, but recommendations include planning during design and implementation, engaging future stakeholders and political support, tailoring the innovation to the context, keeping the innovation as simple as possible, and conducting evaluations [1, 17, 18].

While there is a growing literature related to sustaining and scaling up healthcare innovations generally, little research has focused on translation of dementia-related interventions specifically. The fact that many evidence-based interventions are not transferred into real-world practice deprives people living with dementia and their caregivers of optimal care [19]. A scoping review [4] identified limited research on dissemination, implementation, and sustainment of evidence-based practices in dementia care, with a particular gap in primary care settings where there is increasing demand to provide care for people with dementia and their caregivers. A review of scaling-up strategies in primary care found few studies relevant to these settings [8]. There is also a paucity of evidence on sustaining and scaling dementia care innovations in rural settings [20]. The aging of rural populations [21] combined with increasing risk of dementia with age contributes to growing numbers of people with dementia in rural settings [22], yet the topic remains under-researched [23]. Primary healthcare plays a critical role in rural dementia care due to lack of specialists and the importance of care coordination post-diagnosis [24, 25], thus effective models of primary healthcare for dementia in rural settings are needed [26], along with strategies for translating these into practice and ensuring sustainability. The purpose of this study was to identify factors influencing sustainability and scale-up from the perspective of rural primary healthcare (PHC) providers involved in designing and delivering rural memory clinics.

### The rural primary healthcare memory clinic intervention

**Fig. 1:**
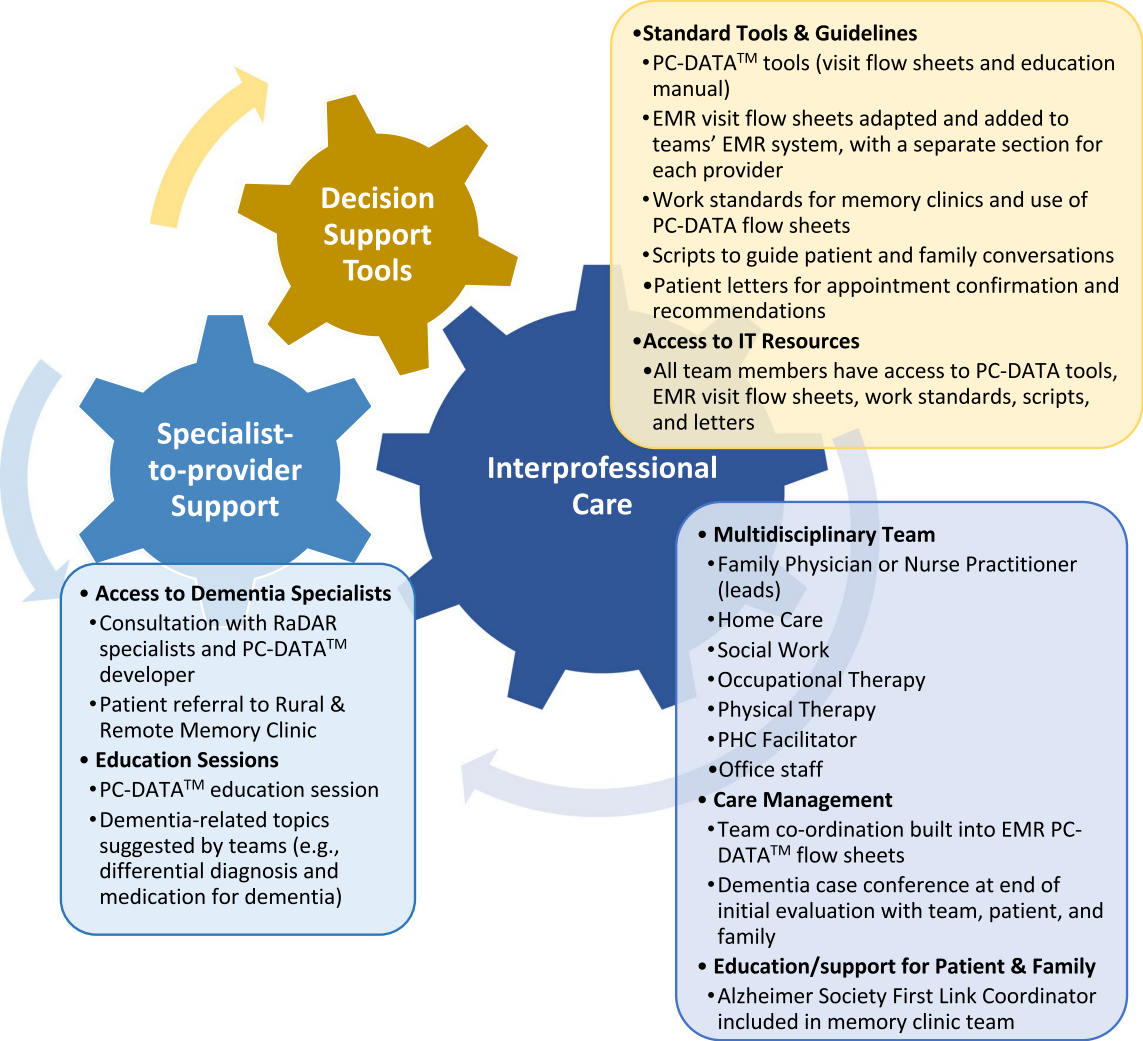
Rural Primary Healthcare Model for Dementia. Note. Configuration of multidisciplinary team depends on availability of providers. RaDAR = Rural Dementia Action Research Team. PC-DATA™ = Primary Care Dementia Assessment and Treatment Algorithm [36]. EMR = electronic medical record. PHC = primary healthcare

For over 20 years the Rural Dementia Action Research (RaDAR) team has conducted a community-based participatory research program focused on health service delivery for people with dementia in rural and remote settings [27], beginning with a university-based specialist memory clinic in 2004 [28–30]. To address challenges in rural dementia care identified in our research [31–33] the RaDAR team developed a conceptual model (Fig. 1) based on a review of evidence-based practices in PHC for dementia that were found to be effective for people living with dementia and care partners [34]. Because of the limited evidence related to PHC for dementia in rural areas, we began collaborating with a rural PHC team (Team 1) to develop an intervention that operationalized the key components of best practices identified in existing literature (represented by the “gears” in Fig. 1) in ways that were feasible, effective, and sustainable in rural contexts. The result was a one-stop interdisciplinary primary care-based memory clinic providing diagnosis and post-diagnostic support for rural-dwelling individuals with dementia and their caregivers (details shown in boxes in Fig. 1). Two patients and their families are assessed at one-day clinics held every 1–2 months in the local PHC clinic or hospital, although the frequency of clinics was reduced during the Covid-19 pandemic. Previous publications have reported on the collaborative process of developing and implementing this intervention [35] and the barriers and facilitators encountered [36]. Once the intervention was fully implemented in Team 1 the focus shifted to sustaining the clinic while scaling to other communities. Memory clinics are now operational in four rural communities within a 280 km radius of Team 1. Outcome studies to evaluate effectiveness of the clinics are now underway, including care partner outcomes and recommendations made by the teams. Initial evaluative data from a study of patient and family experiences has been very positive.

## Methods

### Study design, setting, and participants

This research was conducted in the prairie province of Saskatchewan in western Canada (population 1 million, area 651,000 km^2^, density 1.9 persons/km^2^), in communities located a 4–5 h drive from the University of Saskatchewan where the RaDAR team is located. Thirty-nine percent of Saskatchewan’s population lives in rural areas with less than 10,000 population, compared to 19% in the rest of Canada [37]. Ethics approval was received from the University Behavioral Research Ethics Board and operational approval from the Saskatchewan Health Authority. All study methods were performed in accordance with the relevant guidelines and regulations. A qualitative cross-sectional and retrospective study design was used.

Table 1 describes the four PHC teams, including community size, date of first memory clinic, number of clinics to date, and team composition. Some communities are small (e.g., 337–807 population) but serve a large agricultural catchment area, with the smallest community approximately an hour’s drive to the nearest town. Data were collected through *focus groups* aimed at exploring team member perspectives of factors influencing sustainability and scale-up (cross-sectional data), and a retrospective analysis of *workgroup meetings* held regularly with each team from pre- to post-implementation as part of an ongoing process evaluation. The workgroups were held every one to two months, bringing together the researchers and memory clinic teams to problem-solve implementation challenges and refine the clinic model to fit each new context. A subset of workgroups held in the first year post-implementation for each team were analyzed for references to sustainability and scale-up. Table 2 reports the number of team members involved in the 6 focus groups (*n* = 40) and 16 workgroup meetings (*n* = 100) by team. These numbers represent a total of 38 unique individuals as participants were involved in both focus groups and workgroups. All participants were female except for two males, from two different teams, who participated in the workgroups only. Focus group participants were all female. Table 3 shows the number of focus group and workgroup participants by discipline.

**Table 1:** Community size, date of first clinic, number of clinics to date, and team composition

	Team 1	Team 2	Team 3	Team 4
Community population	1074	10,870	337, 643, 807^a^	1508
Date of first clinic	Dec 2017	Sept 2018	Nov 2018	Feb 2020
Clinics to date	15	14	6	1
Team Composition				
-Family Physician		X		
-Nurse Practitioner	X		X	X
-Occupational therapist	X	X	X	X
-Home care nurse	X	X	X	X
-Social worker		X		
-Physical therapist	X	X	X	X
-Dietitian				X
-Alzheimer Society First Link	X	X	X	X
-Facilitator	X	X	X	X

^a^Team 3 conducts clinics in 3 communities

**Table 2:** Number of focus group and workgroup meetings and participants by team

Meetings	Team 1	Team 2	Team 3	Team 4	Totals
# Focus Groups	2	2	1	1	6^a^
# Workgroups	6	4	4	2	16
Total Meetings	8	6	5	3	22
Participants					
Focus Groups	13	13	6	8	40
Workgroups	39	23	23	15	100
Total Participants	52	36	29	23	140

^a^Separate focus groups to discuss sustaining and scaling the memory clinics were held with two teams and combined for two teams. The 140 participants across focus and workgroups represents 38 unique individuals as the same memory clinic team members were involved in both data collection strategies

**Table 3:** Focus group and workgroup participants by discipline

Participants	Focus Group *n*	Work Group *n*	Total *n*
Family Physician/ Nurse Practitioner	4	15	19
Occupational Therapist	5	14	19
Home Care Nurse	1	12	13
Social Work	6	8	14
Physiotherapist	4	7	11
Dietician	1	2	3
ASOS First Link Coordinator	5	9	14
PHC Facilitator	6	15	21
Manager	8	16	24
MOA/Office Staff	0	2	2
Totals	40	100	140*

*The 140 participants across focus groups and workgroups represents 38 unique individuals as the same memory clinic team members were involved in both data collection strategies

### Data collection and analysis

Six focus groups were conducted by teleconference over a five-month period., using a semi-structured interview guide (Additional file 1) to explore factors influencing sustainability of existing clinics (minimum of 1 year post-implementation) and scale-up (phased expansion) to other rural PHC teams. The focus groups ranged from 31 to 78 min (*M* = 50). Members of longer-running teams had more time to observe influences on sustainability and spread, thus these focus groups were longer compared to newer teams. To accommodate teams’ schedules, separate focus groups were conducted to discuss sustainability and scale-up for two teams and combined focus groups discussing sustainability and scale-up were conducted for two teams. The 16 workgroup meetings (range = 25–116 min, *M* = 50) were held in person (1), by videoconference (2), or by teleconference (13). An interview guide was not used because workgroups were focused on ongoing implementation and operational issues.

The focus groups and workgroups were audio recorded, transcribed, and checked for accuracy. The focus groups were the primary data source; workgroup data provided additional contextual data that were used to verify, refine, and add to the themes. An inductive thematic analysis [38] was used to identify patterns of meaning related to sustainability and scale-up of the clinics. All analytic team members (DM, JK, MB, VE, CC) have been involved in this longitudinal study and attended the focus groups and workgroups. Phases of analysis involved reading and re-reading the data, generating initial codes, collating codes into themes, checking that themes worked in relation to the full data set, refining specifics of the themes, and final analysis including selection of exemplar quotations [38]. Initial coding and theme development was conducted by DM. JK, MB, VE, and CC independently reviewed transcripts and draft themes to refine the analysis.

## Results

The analysis revealed 11 key factors, five that influenced both sustainability and scaling, three related to sustaining, and three related to scaling. These are described below using quotations drawn from the focus groups. Within the transcripts for each focus group participants were numbered consecutively (P1, P2, etc.). Key findings are summarized in Table 4. Over the years of co-developing and implementing the memory clinics in partnership with the PHC teams [35] and conducting an ongoing process evaluation [36] we have initiated various strategies to support implementation and build capacity. Many of these strategies align with the factors identified in this study as having a positive influence on sustaining and scaling and are also reported in Table 4.

**Table 4:** Rural memory clinic team members' perspectives of factors influencing sustainability and spread and associated RaDAR activities

Factors influencing sustaining and spreading memory clinics	How it helps	RaDAR Strategies supporting sustainability and spread of rural PHC memory clinics
BOTH SUSTAIN AND SPREAD		
Positive outcomes for patients & families	-rural communities aging but have limited services -knowing that the clinic is addressing gaps and meeting needs in the community makes teams want to start a clinic and to continue - community awareness that help is available leads to continued referrals -patient and family more comfortable with familiar health care team members -clinics avoid travel/wait times for urban specialists -clinics provide “one-stop shop” with access to all services and provide “wrap-around” support -families feel relief at getting help and less alone and isolated; they know where to go for help -clinics are resulting in earlier diagnosis and management, provision of supports, less stigma	-collecting research data on clinic outcomes that are shared with teams in reports and presentations; reinforces positive impact of the clinics -teams have presented on their clinics and the benefits at the annual RaDAR Rural Dementia Summits and national conferences
Well-developed clinic processes and tools	-clinic has core components but is adaptable depending on available health care professionals -everything needed is ready to use -less daunting to start a clinic -less time investment required by teams than trying to start a memory clinic on their own -standardized process provides structure to follow -reduces uncertainty about what is expected -education sessions build capacity and confidence -clinics are more efficient for some team members	-collaborated with teams to adapt PC-DATA^a^ [39] flow sheets for team-based care in rural context and embed in EMR -created clinic Handbook compiling all clinic resources; provided copy to each team member; updated annually -supported team members to visit specialist memory clinic at university, attend annual RaDAR Summit, and travel with RaDAR to national conferences -researchers shared innovations developed by teams with other teams
Clinic champion and engaged confident leader	-confident, enthusiastic leader acts as a champion for new teams, creating interest and excitement -clinics need a consistent leader who knows the clinic processes and provides direction -clinic leads need skills in both dementia care and leadership in order to lead the implementation	-support clinic leads to develop capacity, e.g., attend dementia conferences, attend RaDAR training sessions
Facilitation and problem-solving	-local PHC facilitator (health region position) helps with communication, scheduling clinics and meetings, developing clinic resources/processes -local facilitators have coordination and quality improvement role, so it is good fit with the clinics -Facilitators and researchers help trouble-shoot and problem-solve to resolve problems early -RaDAR team facilitation keeps teams accountable	-hired Team 1 nurse practitioner part-time to provide leadership and clinical support to teams -hired former Team 1 facilitator living near the teams to attend all clinics, provide operational guidance and collect research data -hold regular workgroup meetings with teams following each clinic to debrief, and problem-solve -hold monthly “check-in” meetings with PHC facilitators and managers to identify/address sustainability issues
Organizational and leadership support	-manager support gives team members permission to engage in developing and practicing in the clinics -access to resources is driven by health system strategic priorities and direction of leadership -health region reorganization underway aligns with the clinic goals of team-based care	-RaDAR research guided by a Steering Committee of all regional Directors and Alzheimer Society leadership -Primary healthcare directors and managers invited to regular team workgroup meetings -connected with provincial health authority leadership to consult about spread strategies
SUSTAIN		
Team passionate and engaged	-commitment and belief in the program help teams collaborate to refine and adapt clinic processes -engagement creates motivation to make the clinic a priority as other demands on teams increase -maintaining a cohesive team requires shared decision-making so all members feel involved -determination to continue during the Covid-19 pandemic shown by adapting to new restrictions	-hold regular workgroup meetings with memory clinic teams and RaDAR researchers to facilitate team communication and decision-making, and keep clinic front of mind
Continuity of team members	-recruitment of healthcare professionals in rural areas can be challenging thus consistency of members helps clinic run smoothly -experienced team members are able to support a new clinic lead while they gain experience	- the clinic handbook is a resource for new members -the EMR manager orients new team members to PC-DATA [36] in the EMR -researchers help orient new facilitators to the clinic -researchers provide 1–1 and group orientations -clinic leads are essential in orientation
Positive outcomes for team members	-team members are more comfortable with each other and working together -collaborations have spread to work outside clinic -team members are more aware of each other’s practice and contributions to dementia care -team members have more information to work with, improving individual/joint recommendations -Alzheimer Society can establish relationship with patient and family to discuss needs and supports -clinics are efficient for some team members because assessments and planning are coordinated in one visit -team approach is gold standard; takes pressure off individual members for issues such as driving	-regular workgroup meetings helps teams to continually improve care coordination -researchers provide regular reports on clinics to the Steering Committee and health authority leadership, underscoring the valuable service teams are providing
SPREAD		
Sustained, successful pilot clinic	-demonstrates that clinic is possible in small rural community and is effective -pilot clinic’s success motivates new teams to start, appeals to team competitiveness -pilot clinic members can engage colleagues in other communities to stimulate interest	-supported development, printing, and distribution of a brochure describing rural PHC memory clinic goals, referral process, assessment process, and benefits. -funded a professional video of a clinic day to raise awareness in community and health care https://www.youtube.com/watch?v=Tzr1MVu7Mpc -memory clinic teams present at annual RaDAR Rural Dementia Summit to share clinic processes, successes
Identify teams with interest, capacity and resources	-focus on teams with interest and resources because both are needed to thrive and survive -let new teams know team composition is flexible -ensure potential new teams are aware of benefits for patients, families, and their own practice -spread is facilitated when there is overlap of team members between new and existing teams	-meet with regional Directors and Managers to identify potential new teams with interest/capacity to start clinic -provide resources to teams where needed to address gaps, such as conference phones, laptops -identifying and engaging teams with interest is part of role of the Team 1 nurse practitioner (hired part-time by RaDAR).
Shadowing and mentoring opportunities	-the most effective strategy for inspiring new team members is to observe a clinic in action to see the value for patients, families, and team members -connecting new members with their counterpart on an existing team helps orient and educate about their role, and provides reassurance	-shadowing and mentoring is built into planned spread strategy: Team 1 nurse practitioner and former team facilitator hired by RaDAR to support teams in starting up and sustaining clinics; new members will be matched with experienced counterpart mentor; opportunity for new members to shadow existing clinics. -delivered initial and ongoing education sessions

^a^*PC-DATA™* Primary Care Dementia Assessment and Treatment Algorithm [39]

### Factors influencing both sustaining and scaling up the memory clinics

#### Positive outcomes for patients and families from the team-based care model

Evidence of the clinics’ effectiveness was critical for sustainability and scale-up. Achieving positive outcomes for patients and families was a key motivation for continuing the clinics, including convenience, timeliness, avoiding multiple visits, and decreasing travel and wait times for specialist appointments. The clinics exemplified the goals of primary healthcare, connecting patients and families with local supports all in one visit.

*P2: It’s a “one-stop-shop.” They come, they do everything at once, and it’s beneficial for families, for clients, that they don’t have to come back again. They get a diagnosis, peace of mind for the family that they get a diagnosis at the end – that what they’re seeing isn’t ‘crazy’. And making a difference in a small-town setting….as opposed to having to wait three or six months to get to [urban centre] to have a CT or to see a specialist, when we can do it all here.* (Team 1)

*P3: We’re working together, we’re pulling departments together for the better of the patient, which is primary healthcare. We’re wrapping the services around the patient, we’re bringing our therapies, our nurse practitioner or physician, the Alzheimer’s Society .... that’s got to be of benefit to the patient because all of those resources are there on that one day.* (Team 3)

The memory clinics filled a gap in services for individuals with memory problems in these aging rural communities, going beyond diagnosis to connect patients and families with supports to enable them to continue care at home longer. Team members valued being able to help where previously they had little to offer.

*P5: It’s all well and good to say, “Yeah, your person has dementia.” But it’s all around services and supports that can help people keep their person at home or keep people safe …. that makes you feel like you’re doing something useful and helpful.* (Team 2)

A key feature of the clinics was bringing together the healthcare team and community-based supports such as the Alzheimer Society. This holistic approach focusing on the patient and family contributed to sustainability because the clinics were meeting identified needs and team members were motivated by being able to offer meaningful support.

*P3: There’s those core pieces that are consistent with all the teams…that team, and that flow, and the connectedness, and engaging with a patient and the family, and giving them the voice – and that respect and dignity to be very involved in the entire process. Connecting with the community [services] as well as healthcare.* (Team 2)

Positive feedback from patients and families reinforced the importance of the clinics in the rural communities and contributed to team member engagement.

*P2: [Sustainability] is many different things…believing in what you’re doing, and believing that this helps patients and families. And hearing patients and families telling us that they appreciate the memory clinics. It’s important work... And we know it’s not going away... When you see that need, how can you not be engaged?* (Team 1)

Other benefits of the clinic for patients and families include being able to get help in their own community from familiar healthcare providers. Memory clinic team members described family members’ relief at getting help, and not being alone and isolated.

*P3: These patients know us so they are comfortable being with [us]. They know all the people that are sitting around helping them make decisions. I think it’s less intimidating for them. And I did see a look of relief on the family’s face when they go out. At least they’ve come, we’ve got things rolling… And the travel – the travel is huge. (Team 1)*

*P6: The main thing that I’ve noticed … was the caregiver’s relief, gratitude, appreciation. Feeling like they’re not alone, that now they’ve got team members to go to. Just relieving that kind of feeling of isolation and – they come in feeling very overwhelmed and very concerned, not knowing what to do. And then leave with something and with people to call.* (Team 2)

Team members involved with longer-running clinics observed that patients are being seen in earlier stages of dementia, and that team members, patients, and families have become more comfortable talking about dementia. Another benefit is that the clinics allow families to come together “to hear the same information and to make a plan.” (Team 4).

*P5: One of the goals of having the clinics was for early detection and diagnosis of dementia. I think the longer the clinics are running the earlier we’re catching folk… And also to decrease the stigma. I really do see a difference in that-- it’s no longer taboo or a subject that we don’t say it.* (Team 4)

#### Well-developed clinic processes and tools

Having the clinic format and resources developed and ready to use supported existing teams in sustaining the clinics and influenced new teams in their decision to start a clinic. The clinic handbook, work standards, education sessions, assessment forms, and support from RaDAR researchers were established, which removed some uncertainty about what was involved. The primary tool was the EMR-based forms for the initial team-based assessment and follow-up appointments that provided a detailed guide to the assessment process. A barrier to scaling up was the fact that some primary healthcare teams in the province use a different EMR platform, thus the flowsheets would need to be adapted.

*P5: It’s not easy, it’s a lot of work but it’s not like reinventing the wheel and starting from scratch. It’s all laid out for you how you set it up and how you do it, so that makes it easier to start a clinic in a town with the support that you get from the other teams. So, that makes it maybe not quite so daunting of a task.* (Team 4).

Coordinating busy team member schedules made booking clinics challenging, but consistently holding them on the same day of the week was helpful. Having a standardized clinic-day agenda with set times for team case conferences and individual team member assessments, specifying where the patient and family are throughout the day, also provided a reassuring format.

*P3: That routine… that standardized process. I find a lot of comfort in that, because when we did step away from the clinic for all those months [due to Covid]… and what did we go to? And with it being fairly standard every time that we come, I find that very comforting, you know, that it’s really quite easy to slip back into what needs to be done.* (Team 2)

Another component of the memory clinics is the initial orientation to assessment forms in the EMR and ongoing education sessions organized by RaDAR on dementia-related topics identified by the teams. These sessions were important for new team members who had limited opportunities to interact with and learn from counterparts in other communities, and the training helped them not feel overwhelmed. Teams identified ongoing education as key to building dementia-specific skills for leaders and members.

*P8: I think coming new into the clinic now…. having the training program would be definitely beneficial because there’s no other OT that’s here on a regular basis for me to talk to about anything… with ever-changing staff, I might not even have somebody to really contact that knows anything about it.* (Team 2)

#### Clinic champion and engaged, confident leader

An engaged leader who is committed to starting a clinic and who acts as a champion for the initiative was essential for launching new clinics. A strong consistent leader who is passionate about the clinic inspires others to want to participate and “keeps propelling it forward” (Team 1).

*P2: Especially to have one strong leader who knows what’s going on, that’s going to stay ...consistent, I think that’s going to make a big difference for starting these memory clinics in other places. And then to lead people through it…. if they’re passionate and knowledgeable, then it will translate to others.* (Team 3)

The leader must have skills in leading others through the day; where this was lacking the clinics struggled. Confidence in dementia diagnosis and management were also important but even when the lead was unsure of the diagnosis the team was still able to provide support and connections to services.

*P5: You really need a strong leader for the team, whether it’s the nurse practitioner or physician, you need to have someone who knows what they’re doing and how to implement it in order to run a clinic. If you don’t have that point of focus then it’s really hard to get anything off the ground.* (Team 2)

#### Facilitation by local facilitators and researchers

The PHC teams involved in the study were supported by permanent health region-funded facilitators who travelled several days a week to each team and were able to incorporate memory clinic activities into their work. They were critical to both sustaining and scaling because of their role in scheduling clinics and meetings, and keeping communication open between the teams and the researchers, and between team members, who are not all co-located except on clinic days.

*P5: Another barrier is that other locations don’t have the primary healthcare facilitator. I know [facilitator] does so much work behind the scenes in scheduling, and getting everyone on board, emailing out all of the agendas …. it would be easy to lose that communication if there isn’t someone kind of always being in the middle, and just being able to be there between the team, between the RaDAR team, and just constantly going back and forth between everyone.* (Team 1)

Another key role of the facilitators was in supporting the clinic itself. They were involved in and knowledgeable about all aspects of clinic development and implementation. They worked with teams to develop additional resources such as referral forms and templates for recommendation letters. On clinic days they coordinated movements of the team, patient, and family, and assisted with troubleshooting.

*P2: I can’t do anything clinically to help any of our professionals. But as far as the rest of the day goes, that’s where we fit into our niches, whether it’s the coordination, the organization, the flow, the problem solving, the improvements. Just helping directing people, be like, “Oh no, no. You go here now.”* (Team 2)

Facilitation by RaDAR researchers also supported sustainability and scale-up. An example is the regular workgroup meetings with the teams to debrief after clinics, discuss solutions to challenges encountered, and plan improvements. On clinic days there was seldom time for these conversations.

*P2: I personally like the accountability of reporting back to your [RaDAR] team. I’ve found that’s been really beneficial…. Having those workgroups on a regular basis, I think it keeps us accountable and keeps the RaDAR clinics in the forefront of all of our minds.* (Team 2)

#### Organizational and leadership support

Interdisciplinary members of the memory clinic teams reported to different managers. Team members with supportive managers felt they had permission to fully engage and commit to being involved in the clinics.

*P2: I think we’re all very fortunate to have a really great, supportive manager too, who let us lead our own way and trust us with managing our time…I can definitely see that in other places potentially being a barrier if they had different or competing priorities for that practitioner time, or the location, or some of those resources.* (Team 2)

Large-scale reorganization of the healthcare system in Saskatchewan, from 13 health regions to one region with 39 networks, began 3 years ago and was still in progress. This change created uncertainty about the impact on programs and jobs, and gaps in some leadership roles occurred while waiting for new positions to be filled. Positive outcomes were also expected because of the focus on team-based primary healthcare.

*P4: I think with the change of primary healthcare encompassing more departments, we’re working more together…. And now moving forward … [all team members] are going to report to the network manager who will be supportive of all the initiatives. You’re not going to have to go to multiple managers to see, ‘can we do this’, it’s going to be part of the team-work.* (Team 1)

Access to adequate resources, including availability of the different healthcare disciplines and adequate space for the clinics, was seen as key to sustaining and scaling the clinics. The interest of healthcare providers was mentioned, since their time is a resource. Alignment of programs with provincial strategic priorities and the vision of leaders can also dictate allocation of limited resources.

*P3: In rural, I think the biggest ability to spread is having those resources in place…. by first asking who’s available, based on their willingness to be involved, ‘cause it’s about time, right? In rural, they wear so many different hats, so if they can also take on a memory clinic, great, we can look at establishing it in a new community.* (Team 2)

### Factors influencing sustainability of the rural memory clinics

#### Team is passionate and engaged

Memory clinic team members noted that commitment to the project and believing that it makes a difference is essential for teams to be able to resolve the inevitable challenges that come up and to manage the ongoing adaptation needed to make it work.

*P3: I always say we learn each time we have a clinic. So everyone is willing to change it up a little bit if this didn’t work or if that didn’t work. Everyone is just so willing to adapt to different scenarios. And I think our team really believes in what we’re doing and I think you really have to have people onboard really believing that what we’re doing is making a difference.* (Team 1)

This engagement creates the motivation to stay involved in the clinic and make it a priority in the face of increasing demands on the healthcare providers involved.

*P3: I have to just reiterate that team willingness and engagement… I think that’s integral for the sustainability of it. Because our workloads are just getting heavier every day, and the demand’s getting higher. But the people that are involved in this project have a passion for dementia care and just have that motivation to continue to provide it. So then it becomes a priority.* (Team 2)

Building and maintaining a committed team requires shared decision-making. Also important is inclusivity and recognition that the support staff are essential team members, communicating with patients and families, making appointments, and tracking the waitlist.

*P2: It’s not just one person makes the decision about how we’re going to do things; we try and involve everybody to help with that decision-making. Then everybody …continues to feel part of the team.* (Team 1)

#### Continuity of team leader and members

Stability of the clinic lead was a key requirement for sustainability. Over the years of developing, implementing, and scaling up the clinics, there has been ongoing turnover of physicians in the primary care settings where the clinics are held. Many physicians in these communities left after 2–3 years of working in the community. It has been difficult to recruit physicians into lead roles in the clinic. There has been much less turnover of nurse practitioners in the RaDAR project area.

*P5: If we’re spreading to any location that doesn’t have nurse practitioners and it’s a physician… there’s a very high likelihood that they’re going to get into the routine of things and then it’s going to be time for them to move on… to sustain, you have to have someone that’s there and going to be there for a while.* (Team 1)

Consistency of memory clinic team members also enhances sustainability. When there is turnover of the lead, the experience of the other team members is critical because they are familiar with clinic operations and they know people in the community who could benefit from the clinic. While the new lead is becoming comfortable in the role, existing team members provide leadership that helps sustain the clinic.

*P2: Our consistency with our homecare staff and with our physio and OT have been super helpful…. Especially when you have a new person…as providers [GP or NP] we’re kind of looked at like maybe we’re supposed to be the leader of it, but when it’s a new provider, that’s not even in our heads. So it’s been so great that other members of our team who are all leaders, more so than myself, stepped up and really were engaged and kept us going. Those members being really resilient and being like, “We have these people [patients]. We need to do this.” That part of it has kept us a little bit more focused and able to keep our clinics going.* (Team 3)

Consistency of team members also helps strengthen professional relationships among team members that carries over outside the clinics.

*P4: I feel the consistency of the teams is really good. It’s the same players at the clinics…. it’s given me the opportunity to get to know some of the other professionals that I can call on them without hesitation.* (Team 3)

#### Positive outcomes for teams and team members

As a result of the interdisciplinary collaboration team members have become more comfortable with each other and developed a shared goal.

*P2: The relationship between the providers, they get to know each other’s scope of practice more. They’re more comfortable with each other for consults about the other patients that aren’t dementia-specific. And I’ve also seen it really give the team a sense of purpose when they’re together. And that’s been really crucial to how successful the clinic has been.* (Team 2)

Team members valued spending more time with each other and becoming more comfortable with each other and with sharing information. The clinic also provides a unique opportunity to learn about other members’ contributions to supporting individuals with dementia and their families.

*P2: Having PT and OT and homecare, and everyone there altogether, I learned so much from listening to [team member] tell me about whatever assessment she did. Obviously I paid a huge amount of attention, but it was so good to see what they’re doing and some of their recommendations they made. I don’t often get to see that.* (Team 3)

Participation of the Alzheimer Society First Link Coordinators also has benefits for everyone. The Coordinators can begin to establish a relationship with patients and families early on when their supports and services can make a real difference. The opportunity for the healthcare providers and Coordinators to develop ongoing relationships has been rewarding and led to consultations outside of the clinic. Another benefit of the interdisciplinary approach is that team members have more information to work with. The informal discussions and case conferences often influence their individual and joint recommendations and increase their confidence in the quality of care they are providing.

*P2: I see it come together when the patient and the family are busy with the other providers and the rest of the providers are still in the room… doing their notes or tasks, or charting, or they’re starting on the recommendation letter…or when social work comes in and being like, “Oh, they said this.” It gives the rest of the professionals… a little bit more information for their assessment and how to best implement recommendations based on what they’re all seeing.* (Team 2)

Despite seeing fewer patients on clinic days, the clinics were more efficient for some team members because all the players were present, streamlining assessment and planning.

*P6: They save time for me to have everybody in the same room, on the same page. And having a really quick plan of action to move forward. Compared to my standard practice… there’s a lot of going back and forth, there’s usually multiple visits to the home. It takes time to follow up with all the other team members. The clinics, for me, are actually way more time saving.* (Team 3)

Team members also valued that decisions were made as a group, especially for challenging issues such as driving cessation that was difficult for some patients to accept. The team approach was seen as a better model of care overall.

*P5: I always talked about OTs getting thrown under the bus for the driving thing because we would get sent a referral and then we were expected to do that. There was a big need and so this is a great way of doing it in a team and really takes the pressure off one person doing all that work. Plus, it’s a better model … as far as all the different clinical pieces.* (Team 4)

Finally, participation in the clinics has helped build capacity in team members, who feel more confident in dementia care management.

*P3: And I guess if I’m going to talk about how this has helped me professionally… I’m way more comfortable now with diagnosis, and knowing how to help them and what to do and what to suggest.* (Team 1)

### Factors influencing scale-up of the rural memory clinics

#### Sustained, successful pilot clinic

For teams considering a clinic, seeing that it was possible to implement and sustain this model in a rural community made the idea of an interdisciplinary primary care memory clinic a reachable goal, “something that could be accessible for them.” (Team 1)

*P2: I found in establishing the clinics that when one site knows about the work that another is doing, that makes them more open and willing to the idea of implementation….I think it brings some tangibility to what a clinic is and that it’s supported…. I think it also appeals to that little bit of competitiveness as well that is like, ‘Well if they can do it, we can do it.’* (Team 2)

Hearing about the sustained success and positive outcomes of the memory clinic inspired new teams to want to provide the same service to their community. Knowing of patients and families in their practices who would benefit motivated them to bring this innovation to their community.

*P2: I think seeing and hearing the success of the other two teams and how it had really taken off and it was of a benefit, there was lots of positive outcomes from them that we thought that it was something that we want to offer our community also.* (Team 4)

Members of existing teams played an important role in scaling up the clinics by engaging with healthcare providers in other communities in the area, describing the benefits of the clinic, and inviting them to observe a clinic.

*P3: They have hired a nurse practitioner in [nearby town]. So she came one afternoon and shadowed me, just to see the EMR. And so I did mention the memory clinic to her and she was quite keen.* (Team 1)

Raising awareness of the clinics in the community helps with sustaining and scaling because patients and families are more likely to ask their healthcare provider about the clinic, and awareness of its benefits may help engage new teams.

*P2: Being and staying visible…I think if the community knows that there’s somewhere they can go for help, that people will keep coming for help… I think it’s up to me to stay visible and make people aware that there is help.* (Team 1)

### Identifying teams with interest, capacity, and resources

Expanding the memory clinics requires identifying potential new PHC teams that are keen to do it, and also have the capacity, in terms of personnel, time, and space. Access to needed resources may be more challenging in rural communities.

*P3: That [adequate resources] is a huge component of success and I mean, I’ve always worked in rural so that’s always forefront in everything we do. If you’re looking at a bigger community, they don’t have the same challenges. So that might not be the first thing they think of.* (Team 2)

Flexibility in team composition was important to communicate so that teams did not rule themselves out prematurely. Although ideally new teams would have a full slate of disciplines involved, current members stressed that parts of the assessment could be covered by others or adapted to fit available configurations of team members and still achieve positive outcomes.

*P2: Some things could be changed or modified… PT can cover some of the OT parts. If teams didn’t have those specific members they can still run. It wouldn’t be as great as having them all, but you could adapt it in ways that I mean would keep the outcome.* (Team 1)

Communicating the benefits of the interdisciplinary approach was identified as a strategy to attract new team members, especially physicians.

*P6: Expressing the benefit, especially if you’re trying to get that practitioner buy-in, saying how it will benefit them as a doctor to have all these different people bringing what they’ve got to the table. Saying, it’ll cut down on this kind of visit or that kind of visit, just the pros of how beneficial it is.* (Team 1)

Starting new clinics in communities close to existing teams had the advantage of cross-over of some team members who covered both communities, which greatly facilitated scale-up.

*P5: Having similar or the same team member in multiple clinics that are nearby, because I found it was really easy to move from [Team 2 to Team 3] because both myself and [team member] were both familiar with how the clinic ran here so we could send that same strategy to [Team 3]. So it made it easy to implement the clinic there.* (Team 3)

#### Opportunity to shadow and be mentored by members of existing clinics

The clinic handbook and other resources were useful to new teams, but team members stated that first-hand experience of team interactions and relationships, and seeing the benefits for patients and families was the most effective way to spark interest.

*P2: There’s so much power in actually being at a clinic and seeing how it works, and seeing how it impacts the patients, seeing how engaged our staff are. That’s really the selling point that I feel can engage more new staff into how valuable our program is.* (Team 2)

Being able to observe a clinic and follow one’s counterpart was a powerful strategy for encouraging new team members to start a clinic, providing reassurance that they have the necessary skills, and creating excitement about their role.

*P5: If I had never shadowed I don’t think I would’ve done it… just going out there [to existing clinic] and seeing that these are tests that we do every day, it’s just using them in a different way. Otherwise, if somebody just asked me to join a dementia team, normally I’d be like, “What do you want me to do?” I think seeing it and seeing that it’s not as complicated as it sounds, will help.* (Team 3)

Connecting new team members with their counterpart on an established team and observing a clinic was helpful for engaging new team members and orienting them to their role.

*P2: The initial meeting that you guys had with us when you were introducing it, I remember being helpful and exciting. Got us excited about it. And then I think that the mentoring and seeing what other teams do, especially if you have a team that’s really good at it, going and watching that or being part of it, or whatever, I think would definitely help.* (Team 3)

Shadowing an existing clinic was identified as a valuable option for getting additional support following an orientation workshop. Connecting new team members with their counterpart on an established team, who could be “on call” for consultation, would also be reassuring to new team members.

*P2: If I would’ve had almost a mentor, mentee type thing for your new clinic. Just to have someone to just say, “Hey, this is what happened, and I don’t know what I should’ve done,” … And even if you didn’t reach out to them, even just to know that you have that backing of someone who has that experience.* (Team 3)

Two strategies for scaling up the clinics were suggested by team members. Expanding one team at a time in nearby communities facilitated shadowing and mentoring because of closer proximity. To expand more quickly, a one-day workshop was suggested.

*P5: If you’re doing a slow spread you could pick a location that’s close to [existing clinic] and then have those providers come down, get some mentorship, go back… and then it spreads slowly away from our corner. But if you’re looking at a fast implementation, I think doing a single-day workshop would be the most beneficial.* (Team 2)

## Discussion

This study addresses a gap in understanding of the factors influencing sustainability and scaling up of innovations in rural dementia care, from the perspective of team members directly involved in developing, implementing, and working in rural PHC memory clinics. Of the 11 factors identified, five influenced both sustainability and scale-up, three were linked to sustainability, and three to scale-up. Overall, these themes relate to team, intervention, and organizational factors. These broad categories align with those reported in other studies (e.g., Stirman et al. [2], Fleiszer et al. [13], Côté-Boileau [40]). However, this study highlighted how the rural context elevates the importance of specific factors that influence sustainability and scale-up, several of which have not been previously identified.

At the *team level*, a committed team champion to galvanize interest and take on a leadership role was critical to both sustaining and scaling, a finding reported by others [26, 41, 42]. Teams looked to physicians and nurse practitioners to lead, but the difficulties of recruiting and retaining physicians in rural communities [43] was a barrier to both sustaining and scaling. Engaging physicians in the memory clinics was a challenge, perhaps because they were uncertain about how long they would stay in the community. Retention of physicians in rural areas in Canada is a long-standing challenge, which has been attributed to practice-based factors (e.g., long working hours, reduced availability of acute care and specialty services, and distance from continuing medical education sessions), sociodemographic factors (e.g., education and employment opportunities for children and spouses, distances from other family members, cultural or language barriers), and higher migration out of rural areas by international medical graduates compared to Canadian and local graduates [43–46]. The fact that all but one of the teams were led by nurse practitioners reflects their relative stability in their communities and greater flexibility in their practice. Our findings are consistent with Yano et al. [18] who reported that champions can support scale-up by sharing their experiences and helping trouble-shoot with new teams. The nurse practitioner leading Team 1 played a major role in raising awareness of the clinic and generating interest among other teams and has been hired on a casual basis by RaDAR as a champion to support implementation in new teams.

In the current study turnover was a challenge because consistency of members helped build team cohesiveness and reduced disruption to clinic operations. The importance of continuity of team members was reported in a review of innovation sustainability in low-resource communities, where staff mobility and turnover were identified as barriers not mentioned in earlier reviews [47]. Misfeldt et al. [48] found more workforce turnover and availability issues among rural and remote PHC teams. In the current study belief in the benefit of the interdisciplinary approach reinforced team member engagement and commitment, helping to counter the challenges of increasing workloads, competing demands of rural practice, and need for continuous adaptation. Although difficult to schedule, ensuring regular opportunities for clinic teams to meet and make shared decisions helped build and maintain commitment. Studies of factors affecting interprofessional primary care team function, including in rural areas, found that teams allocating more time for planning and discussion were better able to tailor the implementation and adapt to a changing context [49], resolve issues [48], and improve collaboration [26]. A review of sustaining and scaling healthcare innovations [40] noted that it is crucial that those involved believe that the innovation improves the quality of care, as their efforts to support the innovation are motivated by the added value they want to achieve. Other strategies for building intrinsic motivation, as outlined in the Sustain and Spread Framework [41], include recognizing the role that each team member plays in improving care and engaging staff in decision-making throughout the change process.

At the *organizational level*, having someone with formal responsibility for facilitating clinic activities was essential for sustaining and scaling. The need for this kind of facilitation has not been reported in previous sustainability studies. McGilton et al. [50] found a need for facilitation, but it was focused on helping staff translate knowledge into practice change at the bedside. Laur et al., who developed the Sustain and Spread Framework [41], found that even after innovations were becoming part of routine practice, effort was still required for “maintaining the routine” [p. 4]. A review of approaches to team-based primary healthcare for dementia in rural settings [26] found that development of strategies for regular communication among team members was necessary for collaborative care. In the current study the rural context and team-based intervention increased the need for organizational support. The clinic involved coordination of multiple healthcare providers from surrounding communities, requiring more assistance with communication, scheduling, and actualizing team decisions that the facilitators provided. A finding from disadvantaged communities [47] was the importance of process factors, including technical assistance and ongoing support from program developers to promote local problem-solving efforts and motivate providers. Participants in the current study reported that regular meetings with the RaDAR team helped with “accountability” and kept the clinics from getting lost amid other priorities. Thus, both internal and external facilitation supported sustainability and scale-up.

Organizational and leadership support have been identified as important for sustaining small rural PHC services [51] and programs in low-resource contexts [47] and were particularly relevant to implementing this team-based intervention in a rural setting. Clinic team members were not normally co-located and were from different disciplines, and therefore reported to different managers. This made engaging and maintaining relationships with leaders more time-consuming and challenging but was critical to ensuring team members felt supported to participate. Identifying teams with interest, capacity, and resources was key to expanding in the rural settings because resources, especially availability of interdisciplinary team members, varied across communities. The intermittent nature of the clinics did not justify hiring additional staff thus the clinics operated with existing personnel who needed to be able to create time in their schedules to participate in the memory clinics, and to have manager support to do so.

The Spread and Sustain framework [41] is based on a study that found scale-up was facilitated by considering local context and readiness, and being responsive to opportunities. In the current study a regional steering committee of managers and directors provided guidance about readiness and capacity among teams in the area, including presence of champions and potential clinic leads, and workforce stability. Health system priorities and structures were also important for resource allocation. Early in the researcher-community partnership, dementia was identified as a regional priority. The provincial health system is currently undergoing reorganization to better support collaborative, team-based care in the community, which aligns with the goals of the memory clinics.

A key *intervention-based* factor was access to well-developed clinic processes and tools, which aligns with earlier recommendations of making the innovation easy to scale [41] and having a model provided [42]. Program familiarity and perceived competence in delivering the program were identified as particularly important in low-resource communities and underlined the need to engage with providers early and to provide continued training [47]. A similar finding was reported in a review of team-based dementia care in rural settings [26]. The importance of capacity-building is reinforced by Coté-Boileau et al. [40] who found that participants’ belief that they are equipped and able to contribute to the program is key to maintaining motivation. Opportunities to observe and be mentored by experienced counterparts emerged as a strong factor in scaling up by providing education about the role and reassuring new team members that they could contribute. Supervision by mentors and practice feedback were found to impact several sustainability elements in under-resourced communities, including program continuation, fidelity, quality of care, and staff retention [47].

A factor that facilitated scale-up was the existence of a sustained and successful pilot clinic, which provided tangible evidence that an interdisciplinary memory clinic was feasible in a rural setting and a model to emulate. This finding links with the concept of “trialability” in the Consolidated Framework for Implementation Research [52], where ability to pilot an intervention provides results that can influence others to move forward with implementation. A successful pilot is also consistent with Diffusion of Innovation Theory [53] where innovators and early adopters demonstrate that the clinic model works and thereby encourages others to follow, and with “observing initial success” in the Sustain and Spread Framework [41]. To ensure that other healthcare providers and communities are aware of the clinic and how it is meeting the needs of patients and families this success must be communicated among potential future teams and the public. A professional video featuring Team 1 was widely shared by the health region and on the town’s Facebook page (see link in Table 4 under Spread), an information brochure was developed, and regular newsletters and reports were shared with regional leaders.

Our experiences in collaborating with rural PHC teams to develop and implement the memory clinics have highlighted the complex nature of the rural healthcare context [51] and are consistent with the view of innovation as an unpredictable journey, and a process of managing and supporting continuous improvement and adaptation [40]. Given that sustainability requires ongoing effort, it is essential to build local capacity to adapt to constant change [14, 54]. In our study clinic team members were able to identify strategies to improve fit of the innovation and manage threats to sustainability, but often needed support from team facilitators, managers, or researchers to follow up or help with execution due to lack of time and competing priorities. With the high workload demands faced by rural healthcare providers, our findings suggest that ongoing support from a designated individual with authority and responsibility for this role will be key to sustainability. Findings related to scaling up reflect team members’ views on what influenced them to participate and what was helpful (and would have been helpful) in getting started, including confidence that the clinic model is feasible and that they have the skills needed to contribute.

### Future research

The RaDAR team has remained engaged with the teams as part of an ongoing process evaluation so at this time it is unclear how clinic sustainability would be affected by withdrawal of the researchers. The teams identified continued facilitation by the RaDAR researchers as helpful, yet some definitions of sustainability refer to continuation of the innovation beyond the research support [9]. The review by Hodge et al. [47] cites the importance of ongoing assistance from program disseminators in low-resource settings, including building local capacity, and ensuring continued support for the innovation before leaving. Further research is needed to explore what such support would look like for the memory clinics, as RaDAR involvement is reduced. Our plans to explore strategies for long-term sustainability and to implement a plan for further scaling up were interrupted by the Covid-19 pandemic. Re-starting the clinics has been hampered by increased workloads and unpredictable re-deployment of team members to pandemic activities. Meetings set up to discuss expansion were disrupted as leaders are overloaded by Covid planning. As pandemic restrictions are gradually being lifted the clinics are re-starting and our research is resuming, informed by the findings of this study. Although not reported as factors by study participants, future research could assess how the presence of other chronic disease clinics and the linkages with other services such as specialist and in-patient care, impact the sustainability and scale-up of the memory clinics. The study communities were relatively homogenous in terms of ethnicity at the time of the study. However, new policies and programs are leading to increased settlement in small and rural towns in Canada [55], thus the impact of greater ethnic diversity on the sustainability and scale-up of rural services such as the memory clincs is also a topic for future research.

### Study strengths and limitations

This study focused on team member perceptions thus findings do not include wider views of managers, directors, and other organizational leaders. Views of clinic patients and family members were not included in this study, but other RaDAR studies are capturing their perceptions of assessment and diagnosis in the clinics, and quality of life and service needs before and after the clinic. The study included a small number of teams but included both cross-sectional and longitudinal data, and perspectives from both newer and more established teams. Team 1 has been sustained since 2017 and Team 2 since 2018, longer than the 2 years suggested as a minimum for defining a sustained program [47, 56]; nevertheless, the longitudinal analysis has been completed over a relatively brief timeframe, and longer-term data are needed. Because the challenges of bringing teams together restricted the time available for the focus groups, the role of sex and gender of the healthcare providers in sustainability and scale-up was not explored in this study. Sex and gender are understudied in all aspects of dementia research [57] but have been shown to influence team dynamics, communication, and outcomes in other health settings [58] and should be explored in future sustain and scale-up studies.

## Conclusion

This study identified factors influencing the sustainability and scale-up of rural PHC memory clinics. Although the challenges of implementing and sustaining healthcare innovations are even greater in low-resource settings [12], our research shows that a PHC intervention for dementia is feasible in a sparsely populated rural area and can be sustained if the right conditions and supports are maintained, including team, organizational, and intervention-based factors. As in other studies we found overlap in a number of factors related to sustainability and scale-up (e.g., Willis et al. [1], Laur et al. [41]). Key factors included the presence of a clinic champion/leader, an engaged and committed team, belief that the clinic is meeting an identified need and making a difference, consistency of team lead and members, facilitation support, and having a ready-to-use intervention model that has been shown to be feasible and effective in a similar setting. Perhaps not surprising given the interdisciplinary intervention, team-based factors were dominant, a finding also reported in a review of interprofessional collaboration in primary care [59]. More time is needed to determine what strategies are required for longer-term continuation of the memory clinics and a larger-scale adoption of the intervention, and this is the focus of our ongoing research.

## Supplementary Information

**Additional file 1.** Focus group interview guide. This semi-structured guide was used for the focus groups conducted with each team. To accommodate teams’ schedules, for Team 1 and Team 2, separate focus groups were conducted to discuss sustaining and scaling up the clinics. For Teams 3 and 4, one focus group was held with each team to discuss both sustaining and scaling the clinics.

## Data Availability

The datasets generated and analyzed during the current study are not publicly available for reasons of participant confidentiality due to location of the primary healthcare teams in small rural communities and small sample size but are available from the corresponding author on reasonable request.

## Abbreviations

RaDAR: Rural Dementia Action Research Team
PHC: Primary healthcare
PC-DATA™: Primary Care: Dementia Assessment & Treatment Algorithm
EMR: Electronic medical record
