# Correction to: Factors infuencing sustainability and scaleup of rural primary healthcare memory clinics: perspectives of clinic team members

**DOI:** 10.1186/s12913-022-07609-y

**Published:** 2022-02-15

**Authors:** Debra Morgan, Julie Kosteniuk, Megan E. O’Connell, Dallas Seitz, Valerie Elliot, Melanie Bayly, Amanda Froehlich Chow, Chelsie Cameron

**Affiliations:** 1 Canadian Centre for Health & Safety in Agriculture, University of Saskatchewan, 104 Clinic Place, Saskatoon, SK S7N 2Z4 Canada; 2 Department of Psychology, University of Saskatchewan, Arts 182, 9 Campus Drive, Saskatoon, SK S7N 5A5 Canada; 3 Department of Psychiatry, Hotchkiss Brain Institute, and O’Brien Institute for Public Health, Cumming School of Medicine, University of Calgary, Room 2919 Health Sciences Centre, 3330 Hospital Drive NW, Calgary, AB T2N 4N1 Canada; 4 School of Public Health, University of Saskatchewan, 104 Clinic Place, Saskatoon, SK S7N 2Z4 Canada

**Correction to: BMC Health Serv Res 22, 148 (2022)**

**https://doi.org/10.1186/s12913-022-07550-0**

Following publication of the original article [1], Fig. 1 was previously replaced with a wrong figure due to a typesetting error. The correct figure is given below.

**Fig. 1:**
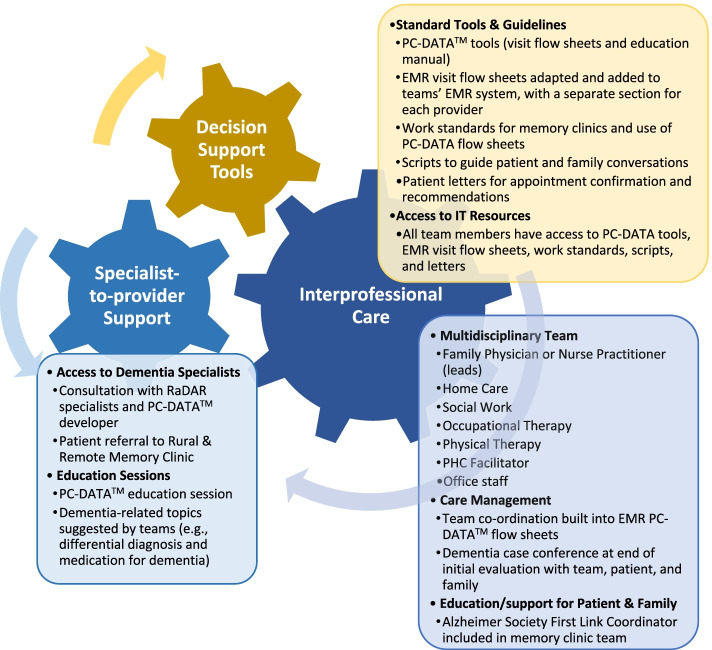
Rural Primary Healthcare Model for Dementia. Note. Configuration of multidisciplinary team depends on availability of providers. RaDAR = Rural Dementia Action Research Team. PC-DATA™ = Primary Care Dementia Assessment and Treatment Algorithm [36]. EMR = electronic medical record. PHC = primary healthcare

The original article has been corrected.
