# Supplementary material for: Factors influencing sustainability and scale-up of rural primary healthcare memory clinics: perspectives of clinic team members
Source: BMC Health Serv Res. 2022 Feb 4;22:148. doi: 10.1186/s12913-022-07550-0 (PMC8814777; doi:10.1186/s12913-022-07550-0)
Supplement: Supplementary file 1 — Additional file 1. Focus group interview guide. This semi-structured guide was used for the focus groups conducted with each team. To accommodate teams’ schedules, for Team 1 and Team 2, separate focus groups were conducted to discuss sustaining and scaling up the clinics. For Teams 3 and 4, one focus group was held with each team to discuss both sustaining and scaling the clinics. [file 12913_2022_7550_MOESM1_ESM.docx]

**Scale-up Questions**

1. What have been the benefits of your memory clinic (for patients, families, community, or yourself professionally)?
2. Thinking about when the clinic model spread to your team, what factors influenced your team’s decision to start a clinic?
3. How can we make it easy for PHC teams to establish a memory clinic in their communities? What was helpful to you and your team in getting started? What was not helpful?
4. If we were to scale-up to *multiple teams* at the same time, what strategies could we use to do that?
5. If time allows: Would the following factors be important for scaling up the rural memory clinics? (From Laur et al., 2018). Being responsive to opportunities, considering local context and readiness, making it easy to spread, being and staying visible, maintaining roles and support new champions

**Sustain Questions**

1. What has helped your clinic continue? What has motivated your team to continue these clinics? What influences sustainability or what is needed to continue the clinics?
2. What are the threats or challenges to sustaining the clinics?
3. When new members join your team, what should be done to orient them to ensure the clinics continue? How important is this for sustainability?
4. What do you think are the core parts of the clinics that can’t be changed? What can be adapted?
5. If time allows: How have the following factors been important for sustaining your memory clinic? Maintaining the new routine, engaging new staff and management, building intrinsic motivation, measuring and reporting, being and staying visible, maintaining roles and support new champions (from Laur et al., 2018).
